# A comprehensive city-level final energy consumption dataset including renewable energy for China, 2005–2021

**DOI:** 10.1038/s41597-024-03529-0

**Published:** 2024-07-07

**Authors:** Guanglei Yang, Guoxing Zhang, Dongqin Cao, Xiulin Gao, Xiaojuan Wang, Shaowen Yang, Pansong Jiang, Donglan Zha, Yuli Shan

**Affiliations:** 1 School of Management, Lanzhou University, Lanzhou, 730000 China; 2 School of Economics, Lanzhou University, Lanzhou, 730000 China; 3 College of Economics and Management, Nanjing University of Aeronautics and Astronautics, Nanjing, 211106 China; 4 School of Geography, Earth and Environmental Sciences, University of Birmingham, Birmingham, B152TT United Kingdom

**Keywords:** Energy economics, Climate-change mitigation

## Abstract

The role of China is increasingly pivotal in climate change mitigation, and the formulation of energy conservation and emission reduction policies requires city-level information. The effectiveness of national policy implementation is contingent upon the support and involvement of local governments. Accurate data on final energy consumption is vital to formulate and implement city-level energy transitions and energy conservation and emission reduction policies. However, there is a dearth of data sources pertaining to China’s city-level final energy consumption. To address these gaps, we developed computational modeling techniques along with top-down and downscaling methods to estimate China’s city-level final energy consumption. In this way, we compiled a final energy consumption inventory for 331 Chinese cities from 2005 to 2021, covering seven economic sectors, 30 fossil fuels, and four clean power sources. Moreover, we discussed the validity of the estimation results from multiple perspectives to enhance estimation accuracy. This dataset can be utilized for analysis in various cutting-edge research fields such as energy transition dynamics, transition risk management strategies, and policy formulation processes.

## Background & Summary

The Paris Climate Agreement and the Sustainable Development Goals include targets at the national level; however, climate change action and nationally determined contributions should be advanced at the city level^1,2^. This commendable solution is already being implemented globally by various organizations, such as the C40 Cities Climate Leadership Group, the International Council for Local Environmental Initiatives (ICLEI), and the United Cities and Local Governments (UCLG)^3–5^. However, without adequate support from local governments, the effective implementation of macro policy goals at the national level, particularly in China, is challenging^6,7^. In fact, in China there are 333 prefecture-level administrative regions, and the straight-line distance between the northernmost city of Shuangyashan (a prefecture-level city in Heilongjiang) and the westernmost city of Kashgar (a prefecture-level city in Xinjiang) is 4,458.78 kilometers. Moreover, these cities are characterized by significant differences in the levels of resource endowment, industrialization, urbanization, and environmental development (Zha *et al*.; Cao *et al*.)^8,9^. By reflecting the internal structural characteristics of provincial-level data that cannot be withdrawn, city-level data offer valuable insights into the intricate interrelationship among different socioeconomic factors at the local level.

Moreover, the radical energy transition fostered by the United Nations Framework Convention on Climate Change (UNFCCC) poses various risks, including an insufficient supply of critical metals^10,11^, employment risks^12,13^, and energy equity risks^14,15^. These risks are predominantly localized in prefecture-level areas. For example, while China’s coal-rich cities of Ordos (a prefecture-level city in Inner Mongolia) and Yulin (a prefecture-level city in Shaanxi) have faced significant unemployment risks due to coal withdrawal, northwestern cities, with abundant wind and solar energy resources, have encountered energy equity challenges^13,16^. Therefore, effective energy and environmental policies must be implemented at the city level. Regrettably, the lack of data on final energy consumption in Chinese cities hampers the effective investigation of city-level energy policies.

Although previous studies did not explicitly focus on city-level final renewable energy consumption, they have encompassed other pertinent city-level factors, particularly CO_2_ emissions. In the existing literature, CO_2_ emissions have been estimated employing two main methodologies: by applying the inversion simulation to nighttime light data^17,18^; and by compiling city-level energy balance tables based on top-down and downscaling approaches^6,19^. As for China, only three studies have compiled city-level energy inventories in China using these two approaches. Chen *et al*.^20^ compiled an energy consumption inventory of 336 Chinese cities from 1997 to 2017 using the inversion simulation method with nighttime light data. Li *et al*.^21^ compiled an energy production inventory of 29 cities in the Central Plains region from 2000 to 2015 based on a top-down approach. Finally, Liu *et al*.^22^ compiled a fossil energy consumption inventory of 286 Chinese cities from 2003 to 2019 using a downscaling method.

The abovementioned studies offer fundamental insights for the estimation of city-level energy inventories; however, certain limitations persist. First, the method of inversion simulation has limitations in providing detailed local information, such as energy structure and end users, as it focuses primarily on total energy consumption at the city level. In addition, estimation results may be affected by factors such as radiation saturation and noise, present in nighttime light data, which may compromise their accuracy^23,24^. Second, although the city-level energy consumption data obtained using top-down and downscaling approaches conform in principle to provincial-level total energy consumption, these studies fail to determine the consumption levels of thermal, nuclear, and renewable energy^21,22^. Consequently, the provided estimates of city-level energy consumption are not comprehensive. Furthermore, there is a dearth of research estimating city-level final energy consumption, which hampers investigations into other crucial aspects such as the energy structure of city-level industries, the clean energy use characteristics of urban and rural residents, and the transition of the power structure.

To fill these knowledge gaps, our study employed top-down and downscaling approaches to estimate the final energy consumption in Chinese cities; the results are illustrated in Table 1. Specifically, our innovative dataset provides comprehensive information on the final energy consumption of 30 fossil fuels (i.e., all energy types listed in the provincial-level energy balance table) and four clean energy sources (i.e., nuclear, hydropower, wind, and solar power) across seven industrial sectors (i.e., those specified in the provincial-level energy balance table) in 331 Chinese cities from 2005 to 2021. This dataset serves as a robust foundation for further analysis of the final energy consumption, the power structure transition, and the emission reduction policies of Chinese cities.

**Table 1:** Studies on city-level energy consumption inventories.

Data level	References	Period	Number of cities	Methods	Types of energy sources	Categories	Items	Accessibility
National	NBSC^25^	1986, 1989, 1991-2022	1	Conservation of energy principle	30	Fossil fuel	Energy balance table	Yes
Provincial	NBSC^25^	1986, 1989, 1991-2022	30	Conservation of energy principle	30	Fossil fuel	Energy balance table	Yes
City	Li *et al*.^21^	2000-2015	29	The top-down approach	20	Fossil fuel	Total energy consumption	No
	Chen *et al*.^20^	1997-2017	336	The inversion simulation method	1	Fossil fuel	Total energy consumption	Yes
	Liu *et al*.^22^	2003-2019	286	The downscaling method	30	Fossil fuel	Total energy consumption	No
	**This study**	**2005-2021**	**331**	**The downscaling method**	**34**	**Fossil fuel & clean power**	**Final energy consumption**	**Yes**

Notes: NBSC represents the *National Bureau of Statistics of China*. According to China’s statistical rules, total energy consumption is categorized into three components: final energy consumption, input and output of transformation, and energy loss.

This paper is structured as follows. The ***Methods*** Section provides details on the data sources and processing methods employed. The ***Data Records*** Section illustrates the evolution trends and spatial characteristics of city-level final energy consumption. The ***Technical Validation*** Section presents a discussion on uncertainty and validity, and the ***Usage Notes*** Section discusses the practical applications of our proposed dataset.

## Methods

### City boundaries and data scopes

In this study, the term ‘cities’ refers to those prefecture-level administrative regions in China that have executive authority above the county level and below the provincial level. Following Liu *et al*.^22^, four municipalities directly under the central government (i.e., Beijing, Tianjin, Shanghai, and Chongqing) are also included in our dataset. This paper presents a comprehensive inventory of final energy consumption in Chinese cities from 2005 to 2021, including data on the final utilization of 34 energy resources across 331 cities (including 327 prefecture-level cities and four municipalities). Significantly, this is the first instance of explicit reporting of clean power consumption at the city level within China. The cities examined in this study encompassed over 98% of the national population, approximately 98% of the national GDP, and around 85% of the land area in 2021, based on NBSC data^25^. Therefore, the sample considered in this study is ideally representative of the entire China.

### Calculation of provincial-level renewable energy consumption

Provincial-level data on renewable energy consumption (i.e., renewable power consumption) from 2015 to 2021 have already been exclusively released by China’s National Energy Administration (CNEA)^26^, see Table S1 in the Supplementary Information. However, it is necessary to reevaluate and recalculate the provincial-level data about renewable power consumption from 2005 to 2014. Unlike other energy sources in China, power production necessitates integration and subsequent redistribution across regional power grids. Consequently, within the same regional grid, the proportion of renewable energy in the total electricity consumption of provinces remains consistent^27^. On this basis, we can distinguish and calculate the consumption of renewable power from overall power consumption. Furthermore, redistribution occurs not only within regional grids, but also within these grids. Therefore, when calculating provincial-level renewable power consumption, it is necessary to consider power transfer into and out of regional power grids.

The State Grid of China is articulated into six distinct regional power grids, covering North China, Northeast China, East China, Central China, Northwest China, and South China. Over the past twenty years, a key strategy, known as the “West-East Power Transmission Project”, has been implemented to effectively address the disparity between electricity supply and demand in China. This Project consists of three main transmission channels: the North Line, the Middle Line, and the South Line. More in detail, the North line supplies power to Northwest China and the North China regional power grids. Its primary function is to transmit the hydropower-generated electricity from the upper reaches of the Yellow River, as well as the thermal power generated in Shanxi and Inner Mongolia, to the Beijing-Tianjin-Tangshan (BTT) region. The Middle Line includes the Central China and East China regional power grids; it facilitates the transmission of hydropower-generated electricity from the Three Gorges and the tributaries of the Jinsha River to meet the energy demands in East China. The South Line includes only the South China regional power grid; it primarily transports hydropower-generated electricity from the Wujiang River in Guizhou, the Lancang River in Yunnan, the Nanpan River, the Beipan River, and the Hongshui River at the Guangxi-Yunnan-Guizhou junctions, as well as the thermal power produced by Yunnan and Guizhou to Guangdong. According to the power transmission channels^28^, China’s six regional power grids will be reintegrated into four regional combined power grids, namely the “Northwest & North China regional power grid”, the “Central China & East China regional power grid”, the “South China regional power grid”, and the “Northeast China regional power grid”. Following Yang *et al*.^28^, the calculation of provincial-level renewable energy consumption involved three sequential steps.

### Step I: Calculation of the exports of renewable power to other provinces

Provincial-level energy balance tables (EBTp) provide detailed statistics on power generation, power exports from other provinces, and renewable power generation (i.e., hydropower, wind power, and solar power). Therefore, the exports of renewable power to other regions can be calculated in the following way:

1 $${RE}{S}_{i}=E{S}_{i}\times \left(\frac{{RE}{P}_{i}}{E{P}_{i}}\right)$$

Where the subscript *i* represents a specific province. ${RES}$ represents the exports of renewable power to other provinces; ${ES}$ represents the power exports to other provinces; ${REP}$ represents renewable power generation; and ${EP}$ represents power generation.

### Step II: Calculation of the imports of renewable power from other provinces

Considering that the renewable energy power imported from other provinces needs to be uniformly distributed through the regional power grid, this calculation needs to follow the transmission and distribution principle of the four regional combined power grids. As already mentioned, the provinces included in the same regional power grid have the same proportion of renewable power. Therefore, the imports of renewable power from other provinces can be calculated in the following way:

2 $${RE}{L}_{i}={\sum }_{i=1\,}^{n}{RE}{S}_{i}\times \left(\frac{E{L}_{i}}{{\sum }_{i=1\,}^{n}E{L}_{i}}\right),1\le n\le {n}_{j}$$

Where ${REL}$ and ${EL}$ represent the imports of renewable power from other provinces and the imports of power from other provinces, respectively. *n*_*j*_ stands for the provinces within the regional combined power grid $j$.

### Step III: Calculation of provincial-level renewable power consumption

The consumption of renewable power at the provincial level (*REC*) is composed of both the generation of renewable power within a province and the inflow of renewable power from other provinces; it was calculated as follows:

3 $${RE}{C}_{i}={RE}{L}_{i}-{RE}{S}_{i}+{RE}{P}_{i}$$

Four types of provincial raw data were required to calculate provincial-level renewable power consumption: power generation, renewable power generation, power exports to other provinces, and power imports from other provinces. Specifically, the power generation data were sourced from NBSC^25^; the data on renewable power generation were obtained from the *China Electric Power Yearbook* (CEPY)^29^; while information on both power exports and imports between provinces was derived from the “energy balance tables” within the *China Energy Statistical Yearbook* (CESY)^30^. Generally, renewable energy mainly exists in the form of power, and consists mainly of hydropower, wind power, and solar power, while other renewable energy sources account for a small share. Accordingly, in 2022, the majority of China’s renewable energy came from hydropower, wind power, and solar power, with other forms contributing less than 5%^31^. The CEPY only reports on these three sources. Therefore, we used these data to represent overall renewable power consumption (for further information, see Yang *et al*.^28^).

To enhance the precision of our estimations, we calibrated them by incorporating the provincial-level renewable electricity consumption data from 2015 to 2021, published by the CNEA^26^. This was done by calculating the deviation ratio between the estimates of this study and the corresponding data from the CNEA from 2015 to 2021. Specifically, for the 2005–2021 estimates, calibration was performed by multiplying the average deviation ratio observed from 2015 to 2021 with the estimated consumption of hydropower, wind power, solar power, and nuclear power.

### Downscaling energy balance tables

#### Traditional provincial-level EBT

The energy balance table (EBT) comprises multiple individual EBTs, generally counted in a matrix form. The EBT allows to intuitively grasp the balanced relationship among energy supply, input & output of transformation, loss, and total final consumption^25^. Therefore, the EBT is China’s most comprehensive source of primary energy data. However, cities still need an EBT, even if its statistical range is limited to national and provincial levels^32^. Traditional provincial-level EBTs include six items (i.e., *Total Primary Energy Supply*, *Input & Output of Transformation*, *Loss*, *Total Final Consumption*, *Statistical Difference*, and *Total Energy Consumption*), covering 30 energy resources such as raw coal, crude oil, natural gas, and electricity. Here, the total energy consumption is equal to the sum of the final energy consumption, the loss, and the input & output of transformation. For example, the total consumption of raw coal is equal to the sum of the final consumption of raw coal, the consumption for the production of secondary energy (i.e., thermal power, thermal, briquette, coking), and the losses of the three parts. To avoid double calculation, we considered only final energy consumption, and excluded intermediate energy consumption and loss for the time being.

#### Extended provincial-level EBT

Conventional provincial-level EBTs provide only overall total consumption, without including detailed breakdowns of power consumption for hydropower, wind, solar, and nuclear power. To fill this gap, we expanded the traditional provincial-level EBTs. Specifically, the provincial-level renewable power consumption was matched with the total power consumption, and the total power consumption was decomposed into five parts: thermal power, hydropower, wind power, solar power, and nuclear power. Accordingly, we added hydropower, wind power, solar power, and nuclear power to the 30 energy resources already included in the traditional provincial-level EBTs, so as to obtain a total of 34 sources.

#### Newly developed city-level EBT

In China, few city-level energy balances exist with multiple time scales. In this study, we queried all EBTs in Chinese cities, and identified two possible cases:

- *Cities with an EBT*. Only a few Chinese cities have released data on final energy consumption for a specific year. For example, Guangzhou has an EBT for the period 2005–2013, and Shenzhen has one for the period 2015–2021. There are several potential areas for improvement in these existing city-level EBTs; in fact, it is not possible to obtain data on a longer time scale and data related to renewable power.
- *Cities without an EBT*. In contrast, the majority cities, including Nanjing, Jinan, Shijiazhuang, and other cities with high energy consumption, do not have an EBT.

It is necessary that existing energy consumption data effectively support more detailed research on energy, especially at the city level. To this purpose, we matched data on renewable power consumption with data on total power consumption, and compiled a city-level EBT including renewable power consumption. The city-level EBT is expected to be a dataset with the most extended time scale, the most comprehensive coverage of city areas, and the most complete energy categories. Following Shan *et al*.^32^ and Liu *et al*.^22^, it was assumed that both cities and their respective provinces exhibit similar levels of sectoral energy intensity and per capita residential energy consumption. On this basis, a recommended approach to link provincial- and city-level final energy consumption is as follows:

4 $${FE}{C}_{{rc}}^{{C}_{k}}={FE}{C}_{{rc}}^{P}\cdot {\alpha }_{r}^{{C}_{k}}$$

Where *r* refers to the *r*-th row in the provincial-level EBT, which represents the activity categories (i.e., “*Agriculture, Forestry, Animal Husbandry and Fishery*”, “*Industry*”, “*Construction*”, “*Transport, Storage and Post*”, “*Wholesale and Retail Trades, Hotels and Catering Services*”, “*Other services*”, and “*Residential consumption*”) of energy consumption. *c* is the *c*-th column in the provincial-level EBT, which represents the energy categories (including 34 energy resources such as fossil energy, hydropower, wind power, solar power, and nuclear power) of energy consumption. ${FE}{C}_{{rc}}^{{C}_{k}}$ and ${FE}{C}_{{rc}}^{P}$ indicate the final energy consumption of type *c* by the *r*-th activity in city *k* and its province *P*, respectively. $\alpha$ is the socio-economic indicator linking provincial and city levels. Specifically, for the ‘industrial sector’ in EBT (i.e., the first 6 sectors in the activity categories), we used the corresponding GDP of each sector to construct index $\alpha$. As for ‘residential consumption’ (i.e., the seventh sector in the activity categories), we employed resident population data to establish the index.

#### Treating discrepancies between provincial and city-level data

In this study, the reasonableness of socio-economic indicators played a decisive role in the accurate compilation of city-level EBTs. To this respect, two critical issues must be addressed during the data processing when utilizing Eq. (4) to downscale provincial data to the city level. First, in a certain province, the socio-economic indicators of some prefecture-level cities may not be available, resulting in a significant difference between the provincial and city-level total. Second, in some specific provinces, some county-level administrative regions are under the direct administration of the provincial level. For example, Hainan includes four prefecture-level cities and 16 county-level administrative units directly under province administration. In these cases, the direct allocation of provincial total data to prefecture-level cities would significantly overestimate the final energy consumption of prefecture-level cities. To address these limitations, two cases were considered in relation to socio-economic indicators linking provincial and city levels:

- **Case A**: No county-level administrative regions are directly under a certain province, and all prefecture-level cities can obtain complete economic and social indicators data. In this case, following Shan *et al*.^33^, we constructed the socio-economic indicators as follows:
  5 $${\alpha }_{r}^{{C}_{k}}=\frac{{V}_{r}^{{C}_{k}}}{{\sum }_{k=1}^{K}{V}_{r}^{{C}_{k}}}$$
  Where ${\alpha }_{r}^{{C}_{k}}$ refers to the socio-economic indicator of Case A. The term *K* represents the number of prefecture-level cities in the target province. ${V}_{r}^{{C}_{k}}$ represents the socio-economic variable of the *r*-th activity category of city *k*. Following the approach used by Liu *et al*.^22^, *V* denotes the corresponding GDP of each industrial sector in EBT; for residential consumption, *V* represents both urban and rural populations.
- **Case B**: The socio-economic indicators of a certain province directly administering county-level administrative regions, or data on the socio-economic indicators of some prefecture-level cities are not available. In this case, following Liu *et al*.^22^, we constructed the socio-economic indicators as follows:

6 $${\alpha }_{r}^{{C}_{k}{\prime} }=\frac{{\sum }_{k=1\,}^{K}{X}_{r}^{{C}_{k}}}{{X}^{P}}\cdot \frac{{V}_{r}^{{C}_{k}}}{{\sum }_{k=1}^{K}{V}_{r}^{{C}_{k}}}$$

Where ${\alpha }_{r}^{{C}_{k}{\prime} }$ refers to the socio-economic indicator of Case B. The terms ${X}_{r}^{{C}_{k}}$ and *X*^*P*^ represent the GDP or resident population of the *r*-th activity category of city *k* and the corresponding data of the province where the city is located, respectively.

## Data Records

The comprehensive dataset built in this study is available for download on *Figshare*^34^. The dataset encompasses a total of 61,897 records, covering the final energy consumption of 34 energy sources across 327 prefecture-level cities and four municipalities in China. The unit is labeled as 10,000 tons of standard coal, and the temporal scope spans from 2005 to 2021. Among these records:

- 5,627 records are for city-level final energy consumption (331 cities, 2005–2021);
- 5,627 records are for city-level final energy consumption of coal total (331 cities, 2005–2021);
- 5,627 records are for city-level final energy consumption of all petroleum products (331 cities, 2005–2021);
- 5,627 records are for city-level final energy consumption of natural gas (331 cities, 2005–2021);
- 5,627 records are for city-level final energy consumption of thermal power (331 cities, 2005–2021);
- 5,627 records are for city-level final heat consumption (331 cities, 2005–2021);
- 5,627 records are for city-level final other energy consumption (331 cities, 2005–2021);
- 5,627 records are for city-level final energy consumption of hydropower (331 cities, 2005–2021);
- 5,627 records are for city-level final energy consumption of wind power (331 cities, 2005–2021);
- 5,627 records are for city-level final energy consumption of solar power (331 cities, 2005–2021); and
- 5,627 records are for city-level final energy consumption of nuclear power (331 cities, 2005–2021).

The final energy consumption includes 34 types of energy resources. The coal category encompasses 11 forms of coal-related energy, such as raw coal and coke. Similarly, there are 14 varieties of petroleum-related energy, such as crude oil and gasoline. Since 2005, to prevent data fraud and duplicated statistics by local governments, the NBSC has implemented a nationwide unified system for basic statistical reporting^35^. This ensures that the statistical caliber of data records after 2005 remains relatively consistent, and allows for meaningful comparisons between statistical results. This dataset covers 327 prefecture-level cities and four municipalities; accordingly, this study selected 2005 as the starting year for analysis. The Supplementary Information provides more detailed spatiotemporal characteristics of city-level final energy consumption in China.

## Technical Validation

### Uncertainty analysis

In this study, potential uncertainties existed in relation to three aspects. **First**, we roughly divided the six regional power grids into four regional combined power grids to estimate provincial-level renewable power consumption, disregarding inter-provincial transmission within a same regional power grid and between different regional power grids. Although the power transmission of these parts is relatively small, a certain degree of bias was anyway introduced in the estimation of provincial-level renewable energy consumption. **Second**, the downscaling approach implies that provinces and their cities have identical energy intensity and energy consumption per capita. However, due to differences in economic development level, technology level, and energy structure among provinces and their cities, there are variations in energy intensity and energy consumption per capita. Therefore, uncertainty arises when calculating city-level final energy consumption using Eqs. (4–6). **Third**, socio-economic indicators were constructed using data on the GDP of the six major industries and the values of urban and rural population; however, these data are only occasionally complete. For instance, for prefecture-level cities in Fujian, it is possible to obtain only GDP data for the tertiary industry but not detailed data for transport, storage and post services, wholesale and retail trade, and hotels and catering services. Consequently, we must use tertiary industry GDP to construct socio-economic indicators under such circumstances, increasing the uncertainty in calculation results.

### Validation of provincial-level renewable energy consumption data

**Fig. 1:**
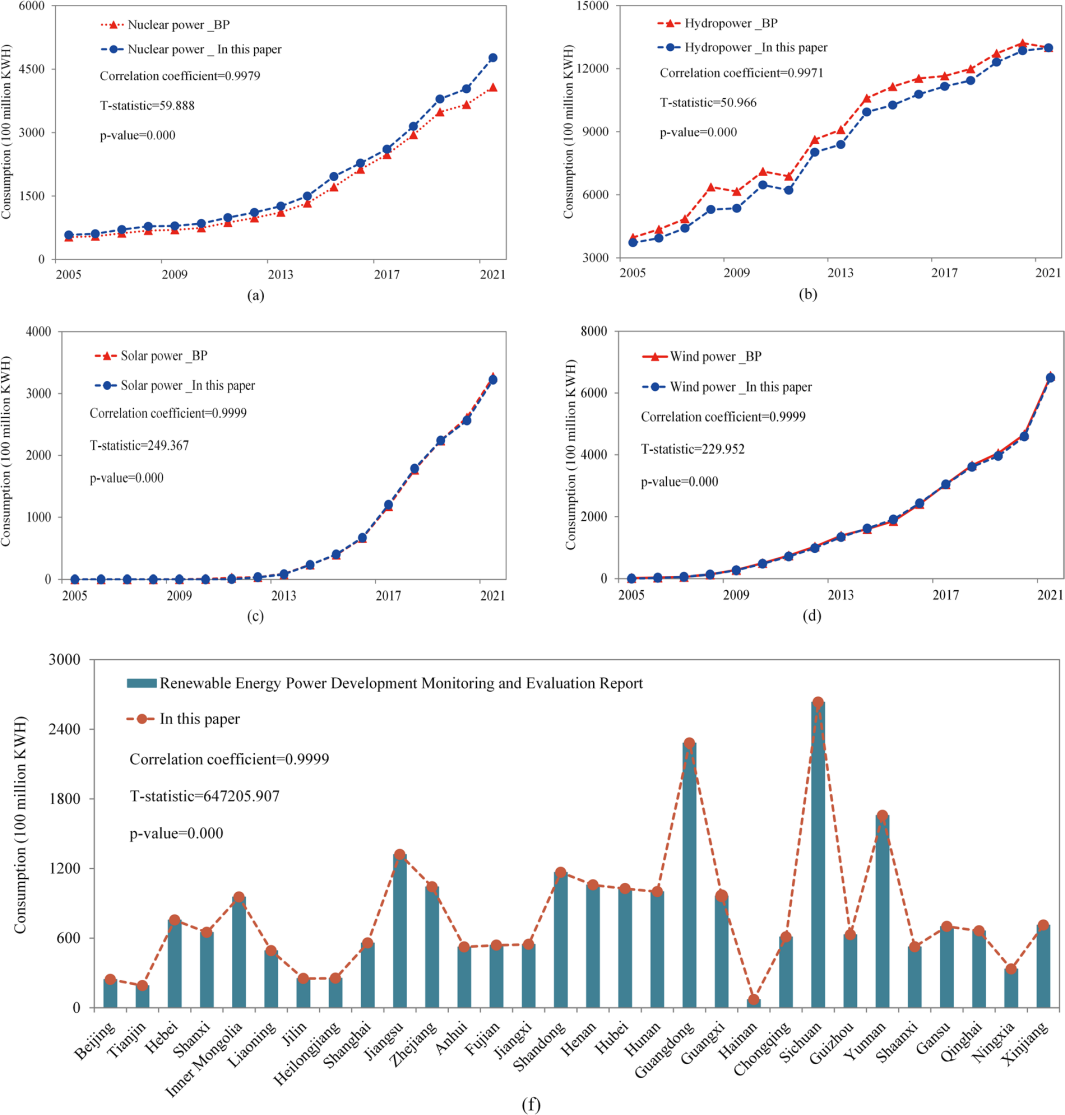
Comparison between the results of this study and the data of the *BP Statistical Review of World Energy* and the *REPDMER*. (**a**) nuclear power, (**b**) hydropower, (**c**) solar power, (**d**) wind power, and (**f**) provincial-level renewable energy consumption in 2021.

This study substantiated the reliability of regional estimates on renewable energy consumption. First, we compared the estimates of China’s consumption of nuclear power, hydropower, solar power, and wind power for the period 2005–2021 (aggregated at the city level) with the corresponding data from *BP Statistical Review of World Energy*^31^. The comparative results are illustrated in Fig. 1(a–d). Despite slight disparities regarding nuclear power and hydropower consumption, both estimates exhibit similar trends over time, as well as comparable rates of marginal growth. Moreover, the correlation coefficients for all the four categories of clean power exceed 0.99, indicating a strong and statistically significant relationship at the 1% level. Second, we compared our estimates of renewable energy consumption across provinces with data from the *Renewable Energy Power Development Monitoring and Evaluation Report* (the REPDMER)^36^. Remarkably, both methodologies exhibit a remarkable degree of similarity in terms of renewable energy consumption patterns, with provinces consistently maintaining their respective rankings (see Fig. 1(f)). Hence, substantial evidence confirms the credibility and accuracy of our provincial-level estimates of renewable energy consumption.

### Validation of economic significance

Final energy consumption is primarily driven by economic activities and residential life, excluding the consumption and loss of secondary energy used for processing and conversion. As such, it is closely associated with both economic output and population. Table 2 presents the Pearson correlation coefficients between estimated city-level final energy consumption and various city-level economic indicators. The average Pearson correlation coefficients between city-level final energy consumption (FEC), on the one side, and gross domestic product (GDP) and resident population (POP), on the other side, from 2005 to 2021 were 0.854 and 0.833, respectively. Furthermore, the average Pearson correlation coefficients between city-level FEC and GDP in the three industries were equal to 0.780, 0.832, and 0.879, respectively. It should also be noted that all coefficients fluctuate between 2% and 8%, indicating high stability in the estimation of city-level final energy consumption.

**Table 2:** The Pearson correlation coefficients between the estimated results and economic indicators.

Year	All cities	All cities	Primary industry	Secondary industry	Tertiary industry
	FEC vs. GDP	FEC vs. POP	FEC vs. GDP	FEC vs. GDP	FEC vs. GDP
2005	0.878^c^	0.801^c^	0.801^c^	0.863^c^	0.917^c^
2006	0.878^c^	0.820^c^	0.791^c^	0.861^c^	0.909^c^
2007	0.885^c^	0.811^c^	0.810^c^	0.870^c^	0.909^c^
2008	0.890^c^	0.833^c^	0.786^c^	0.888^c^	0.887^c^
2009	0.898^c^	0.809^c^	0.777^c^	0.895^c^	0.888^c^
2010	0.894^c^	0.777^c^	0.778^c^	0.891^c^	0.881^c^
2011	0.887^c^	0.803^c^	0.794^c^	0.886^c^	0.868^c^
2012	0.884^c^	0.811^c^	0.797^c^	0.884^c^	0.863^c^
2013	0.864^c^	0.838^c^	0.790^c^	0.859^c^	0.865^c^
2014	0.853^c^	0.849^c^	0.798^c^	0.851^c^	0.841^c^
2015	0.862^c^	0.850^c^	0.803^c^	0.846^c^	0.852^c^
2016	0.840^c^	0.844^c^	0.770^c^	0.810^c^	0.863^c^
2017	0.843^c^	0.855^c^	0.724^c^	0.817^c^	0.857^c^
2018	0.807^c^	0.861^c^	0.779^c^	0.754^c^	0.881^c^
2019	0.787^c^	0.875^c^	0.773^c^	0.716^c^	0.894^c^
2020	0.774^c^	0.873^c^	0.753^c^	0.710^c^	0.887^c^
2021	0.789^c^	0.851^c^	0.737^c^	0.742^c^	0.880^c^
Mean	0.854	0.833	0.780	0.832	0.879
Coefficients of variation.	0.048	0.033	0.030	0.076	0.024

Note: Superscript c represents the 1% significance level.

### Validation of estimation accuracy

A thorough investigation of existing literature confirmed that there is a dearth of data on China’s city-level final energy consumption. On the other side, a strong correlation exists between fossil energy and CO_2_ emissions. In 2022, China’s CO_2_ emissions from fossil fuels reached 10.442 billion tons, accounting for over 91% of the total CO_2_ emissions^37^. Building upon this observation, we established a correlation between estimated final fossil energy consumption (FFEC), encompassing coal, oil, gas, and thermal power, and the city-specific CO_2_ emissions data taken from reputable sources such as the Open-source Data Inventory for Anthropogenic CO_2_ (ODIAC)^38^ and the China Emission Accounts and Datasets (CEADs)^39^. In the former, city-level CO_2_ emission estimates are derived through inverse simulation using nighttime light data, while in the latter, top-down methods are employed for downscaling purposes. This study selected 26 provincial capitals as representative cities; the Pearson correlation coefficients are presented in Table 3. The estimated city-level final fossil energy consumption exhibited a strong positive correlation with the CO_2_ emissions data from the ODIAC and CEADs datasets. In both sets of results, the Pearson correlation coefficients of all capital cities exceeded 0.65, with 20 out of 26 cities surpassing 0.85. These findings indicate a high consistency between the estimated data and existing records.

**Table 3:** The Pearson correlation coefficients between final fossil energy consumption and CO_2_ emissions of ODIAC and CEADs.

Cities	FFEC vs. ODIAC	FFEC vs. CEADs	Cities	FFEC vs. ODIAC	FFEC vs. CEADs
Hefei	0.924^c^	0.919^c^	Nanjing	0.945^c^	0.920^c^
Fuzhou	0.990^c^	0.985^c^	Nanchang	0.894^c^	0.940^c^
Lanzhou	0.936^c^	0.877^c^	Shenyang	0.890^c^	0.937^c^
Guangzhou	0.945^c^	0.971^c^	Hohhot	0.847^c^	0.914^c^
Nanning	0.916^c^	0.979^c^	Yinchuan	0.892^c^	0.970^c^
Guiyang	0.910^c^	0.867^c^	Xining	0.861^c^	0.910^c^
Haikou	0.963^c^	0.850^c^	Jinan	0.797^c^	0.933^c^
Shijiazhuang	0.950^c^	0.927^c^	Taiyuan	0.732^c^	0.652^b^
Zhengzhou	0.915^c^	0.972^c^	Xi’an	0.975^c^	0.951^c^
Harbin	0.835^c^	0.869^c^	Chengdu	0.981^c^	0.982^c^
Wuhan	0.877^c^	0.933^c^	Urumqi	0.932^c^	0.858^c^
Changsha	0.901^c^	0.917^c^	Kunming	0.891^c^	0.935^c^
Changchun	0.803^c^	0.924^c^	Hangzhou	0.972^c^	0.810^c^

Note: Superscript c and b represent the 1% and 5% significance levels.

### Limitations and future work

The datasets employed in this study are inevitably subject to certain limitations; nevertheless, we are dedicated to enhance our future research efforts along the following way:

- In this study, we considered only data on final energy consumption, excluding data on energy supply, transformation, and loss. To address this limitation, in future research we aim to broaden the statistical scope by compiling a comprehensive city-level EBT encompassing six items (i.e., the same items in the provincial-level energy balance table).
- We assumed that cities within the same province have identical values of sectoral energy intensity and energy consumption per capita. However, variations in economic indicators exist among cities within the same province. Therefore, in future research, we plan to optimize computational modeling techniques and the top-down approach by incorporating city-level heterogeneity characteristics.
- In this study, renewable energy consumption includes hydropower, wind power, and solar power, excluding biomass, hydrogen, and ocean energy because of their small quantities of consumption and the absence of reliable data. Therefore, our future research will expand the scope to include these types of renewable energies.
- The downscaling method utilized in this paper is based on the establishment of a socio-economic indicator that links provinces and cities. It should be noted that not all cities have access to GDP data for the six major industrial sectors. In such cases, we can only utilize the GDP of the three industries as an approximation for constructing socio-economic indicators. Therefore, our objective in future research is to enhance sub-industry fundamental data through computational modeling techniques.

## Usage Notes

This study presents a comprehensive dataset on final energy consumption across 331 Chinese cities from 2005 to 2021. The dataset, along with 11 separate data tables on fossil fuels and clean energy, will be annually updated. Based on the methodologies and data employed in this study, these datasets are replicable, auditable, and scalable. All data have been uploaded to *Figshare* for unrestricted access. Furthermore, these datasets offer valuable insights for practical applications in energy economics, transition risk management, and policy formulation. These findings are particularly significant due to the following reasons:

- The dataset proposed in this study outlines the comprehensive characteristics of China’s city-level final energy consumption, as including thermal, hydropower, wind, solar, and nuclear power. Compared to existing data, our dataset allows a comprehensive observation of the spatio-temporal patterns of final energy consumption in Chinese cities. This facilitates an in-depth analysis of the factors driving final energy consumption in city environments, thereby contributing to a better understanding of the subject.
- The dataset proposed in this study is compatible with other social data, applicable to cross-sectional, time-series, and panel data, thereby facilitating empirical research on the economic, social, and environmental external risks arising from city-level energy development. For instance, this dataset enables to assess the historical contributions of city-level energy transitions towards carbon emissions reduction, as well as the supply risks associated with crucial mineral resources during the transition to a clean power system.
- The dataset proposed in this study includes city-level data on fossil and renewable energy, thus enabling the assessment of the effectiveness of energy and environmental policies at the city level. For example, it enables the assessment of whether the “time-of-use tariff” policy can effectively reduce city-level electricity consumption, whether the “low-carbon pilot city” policy positively impacts thermal power energy efficiency, and whether the “coal to electricity” policy significantly contributes to clean energy transition in Chinese cities.

### Supplementary information

provincial-level renewable energy consumption and city-level final energy consumption

The code of Table 2

The data of Table 2

The code of Table 3

The data of Table 3

## Data Availability

The supplementary information provides access to the pertinent code and data employed for computation and analysis in this study.
