# Supplementary material for: A comprehensive city-level final energy consumption dataset including renewable energy for China, 2005–2021
Source: Sci Data. 2024 Jul 7;11:738. doi: 10.1038/s41597-024-03529-0 (PMC11228046; doi:10.1038/s41597-024-03529-0)
Supplement: Supplementary file 1 — provincial-level renewable energy consumption and city-level final energy consumption [file 41597_2024_3529_MOESM1_ESM.docx]

**Supplementary Information**

**Renewable energy consumption at the provincial level in China**

**Table S1** The consumption of renewable energy at the provincial level in China (unit:100 million kilowatt hours).

| Provinces | 2015 | 2016 | 2017 | 2018 | 2019 | 2020 | 2021 |
| --- | --- | --- | --- | --- | --- | --- | --- |
| Beijing | 81 | 107 | 130 | 151 | 148 | 187 | 244 |
| Tianjin | 63 | 75 | 89 | 98 | 112 | 141 | 191 |
| Hebei | 260 | 335 | 401 | 448 | 530 | 559 | 756 |
| Shanxi | 152 | 218 | 280 | 355 | 417 | 440 | 649 |
| Inner Mongolia | 354 | 428 | 554 | 624 | 672 | 821 | 954 |
| Liaoning | 197 | 259 | 260 | 326 | 371 | 418 | 492 |
| Jilin | 105 | 138 | 156 | 187 | 196 | 244 | 252 |
| Heilongjiang | 119 | 141 | 188 | 189 | 219 | 238 | 254 |
| Shanghai | 419 | 484 | 509 | 503 | 542 | 561 | 558 |
| Jiangsu | 599 | 699 | 856 | 903 | 941 | 1,072 | 1,321 |
| Zhejiang | 658 | 772 | 809 | 827 | 946 | 946 | 1,042 |
| Anhui | 157 | 221 | 274 | 317 | 363 | 427 | 524 |
| Fujian | 504 | 704 | 511 | 440 | 577 | 473 | 539 |
| Jiangxi | 266 | 322 | 328 | 327 | 392 | 410 | 546 |
| Shandong | 270 | 327 | 399 | 584 | 727 | 860 | 1,167 |
| Henan | 261 | 311 | 461 | 578 | 692 | 731 | 1,058 |
| Hubei | 616 | 678 | 803 | 788 | 721 | 927 | 1,026 |
| Hunan | 672 | 743 | 798 | 735 | 828 | 909 | 1,000 |
| Guangdong | 1,758 | 1,902 | 1,932 | 2,080 | 2,308 | 2,294 | 2,281 |
| Guangxi | 770 | 678 | 744 | 783 | 824 | 878 | 964 |
| Hainan | 26 | 35 | 41 | 44 | 52 | 59 | 71 |
| Chongqing | 432 | 447 | 488 | 512 | 529 | 610 | 610 |
| Sichuan | 1,575 | 1,745 | 1,842 | 2,013 | 2,139 | 2,344 | 2,633 |
| Guizhou | 450 | 512 | 493 | 537 | 549 | 646 | 629 |
| Yunnan | 1,233 | 1,147 | 1,317 | 1,400 | 1,503 | 1,634 | 1,657 |
| Shaanxi | 163 | 135 | 240 | 324 | 379 | 434 | 525 |
| Gansu | 466 | 457 | 546 | 625 | 696 | 722 | 701 |
| Qinghai | 461 | 398 | 446 | 577 | 586 | 629 | 662 |
| Ningxia | 137 | 187 | 225 | 268 | 280 | 277 | 334 |
| Xinjiang | 362 | 410 | 521 | 574 | 631 | 652 | 712 |

**Spatiotemporal characteristics of city-level final energy consumption**

The abovementioned datasets provide us with more detailed and comprehensive insights into the energy consumption characteristics in Chinese cities. **Figure S1** illustrates the final energy consumption and its composition for 327 cities in China, revealing distinct group characteristics at the city level. Notably, North China, East China, and South China exhibited significantly higher levels of final energy consumption than other regions. Moreover, the final energy consumption of the majority of provincial capitals and economically developed cities surpass 38 million tons of standard coal (**see Figure S1 (a)**). In the period from 2005 to 2021, a gradual decline was recorded in the proportion of coal total consumption, while petroleum products showed signs of stabilization. In contrast, the consumption of natural gas, electricity, and other forms of energy witnessed varying degrees of increase (**see Figure S1 (b)**). These findings indicate that China’s overall energy structure has been gradually transitioning towards lower carbon intensity.

**Figure S1** City-level final energy consumption in China (unit: ten thousand tons of coal equivalents). (a) the spatial distribution in 2021, and (b) the composition in 2005-2021.

**Spatiotemporal characteristics of city-level final** **fossil energy consumption**

The spatial distribution of the final consumption of coal (total), petroleum products (total), natural gas, and thermal power in 2021 is illustrated in **Figure S2**. A high total coal consumption was observed in North China, particularly in provinces such as Hebei, Inner Mongolia, Shanxi, and other regions abundant in coal resources. Conversely, the cities south of the Yangtze River exhibited relatively lower levels of total coal consumption, especially in Southwest and South China (see **Figure S2 (a)**). The economically developed coastal areas and provincial capitals were characterized by high petroleum products (total), while the northwest region had a significantly lower consumption of petroleum products consumption than other areas (see **Figure S2 (b)**). Cities with a high consumption of natural gas were primarily concentrated in North China, East China, Sichuan, and Guangdong. In contrast, the majority of the cities located in Central China displayed relatively low levels of natural gas consumption (see **Figure S2 (c)**). Compared to the other three primary fossil energy sources, thermal power consumption exhibited more distinct regional characteristics. The cities with a high consumption of thermal power were mainly located in North China, Eastern China, and Guangdong; in contrast, cities in Southwest and Northwest China consumed considerably less thermal power than those in Central and Eastern China (see **Figure S2 (d)**).

**Figure S2** The fossil energy final consumption of Chinese cities in 2021 (unit: ten thousand tons of coal equivalents). (a) coal total, (b) petroleum products total, (c) natural gas, and (d) thermal power.

**Spatiotemporal characteristics of city-level final** **clean energy consumption**

The spatial distribution and composition of clean power consumption in 327 Chinese cities are illustrated in **Figure S3**. Significant regional disparities exist in clean power consumption among these cities. Specifically, only three Southwest and South China cities consumed more than 5.50 million tons of clean power. Clean power consumption remained below 1.45 million tons of standard coal in 269 out of 327 cities (see **Figure S3 (a)**). While hydropower dominated as the largest source of clean power consumption, its proportion within the overall mix declined annually since 2005. This decline has been offset by increased wind and solar power consumption (see **Figure S3 (b)**).

**Figure S3** City-level clean power final consumption in China (unit: ten thousand tons of coal equivalents). (a) the spatial distribution in 2021, and (b) the composition in 2005-2021.

The spatial distribution of hydropower, wind, solar, and nuclear power consumption in 327 Chinese cities in 2021 is illustrated in **Figure S4**. Due to the apparent disparities in regional power resource endowments, significant variations were observed in the spatial distribution of clean power consumption. In general, hydropower consumption was considerably higher in Southwest, Central, and Southern China, while the majority of cities in North and Northeast China consumed less than 0.36 million tonnes (see **Figure S4 (a)**). The cities with a high consumption of wind power were primarily located north of the Yangtze River, whereas the majority of cities in Southwest, Central, and South China exhibited low consumption (see **Figure S4 (b)**). Interestingly, the spatial distribution characteristics of solar power consumption closely resemble those of wind power consumption (see **Figures S4 (c)**). The cities with a significant consumption of nuclear power were predominantly situated in the coastal areas of China. In contrast, nuclear power consumption in central and western regions was generally low, with less than 0.10 million tonnes recorded in 237 cities (see **Figure S4 (d)**).

**Figure S4** The clean power final consumption of Chinese cities in 2021 (unit: ten thousand tons of coal equivalents). (a) hydropower, (b) wind power, (c) solar power, and (d) nuclear power.
