# Supplementary material for: A comprehensive city-level final energy consumption dataset including renewable energy for China, 2005–2021
Source: Sci Data. 2024 Jul 7;11:738. doi: 10.1038/s41597-024-03529-0 (PMC11228046; doi:10.1038/s41597-024-03529-0)
Supplement: Supplementary file 2 — The code of Table 2 [file 41597_2024_3529_MOESM2_ESM.docx]

***Table 2***

use "C:\Users\PC\Desktop\城市能源平衡表编制\退修\code and data\相关系数数据.dta", clear

**Results in Column (2)**

local path "C:\Users\PC\Desktop\Correlation coefficient\\results(all cities_FEC vs GDP).txt"

file open myresults using `"`path'"', write replace

local sumr 0

local count 0

forvalues y = 2005 / 2021 {

quietly sum gdp_city if year == `y'

local N = r(N)

quietly correlate gdp_city ec_city if year == `y'

local r = r(rho)

local sumr = `sumr' + `r'

local count = `count' + 1

local t = `r' * sqrt(`N'-2) / sqrt(1-`r'^2)

local df `N' - 2

local p = 2 * (1 - ttail(`df', abs(`t')))

file write myresults "Year `y':\t r = `r',\t t = `t',\t p = `p'" _n

}

local meanr = `sumr' / `count'

local sumsq 0

forvalues y = 2005 / 2021 {

quietly correlate gdp_city ec_city if year == `y'

local r = r(rho)

local sumsq = `sumsq' + (`r' - `meanr')^2

}

local sdr = sqrt(`sumsq' / (`count' - 1))

local cvr = (`sdr' / `meanr') * 100

di "Average correlation coefficient: `meanr', Coefficient of variation: `cvr'%"

file write myresults "Average correlation coefficient: `meanr', Coefficient of variation: `cvr'%" _n

file close myresults

**Results in Column (3)**

local path "C:\Users\PC\Desktop\Correlation coefficient\\results(all cities_FEC vs POP).txt"

file open myresults using `"`path'"', write replace

local sumr 0

local count 0

forvalues y = 2005 / 2021 {

quietly sum ec_pop if year == `y'

local N = r(N)

quietly correlate ec_pop pop_city if year == `y'

local r = r(rho)

local sumr = `sumr' + `r'

local count = `count' + 1

local t = `r' * sqrt(`N'-2) / sqrt(1-`r'^2)

local df `N' - 2

local p = 2 * (1 - ttail(`df', abs(`t')))

file write myresults "Year `y':\t r = `r',\t t = `t',\t p = `p'" _n

}

local meanr = `sumr' / `count'

local sumsq 0

forvalues y = 2005 / 2021 {

quietly correlate ec_pop pop_city if year == `y'

local r = r(rho)

local sumsq = `sumsq' + (`r' - `meanr')^2

}

local sdr = sqrt(`sumsq' / (`count' - 1))

local cvr = (`sdr' / `meanr') * 100

di "Average correlation coefficient: `meanr', Coefficient of variation: `cvr'%"

file write myresults "Average correlation coefficient: `meanr', Coefficient of variation: `cvr'%" _n

file close myresults

**Results in Column (4)**

local path "C:\Users\PC\Desktop\Correlation coefficient\\results(primary industry_FEC vs GDP).txt"

file open myresults using `"`path'"', write replace

local sumr 0

local count 0

forvalues y = 2005 / 2021 {

quietly sum gdp_primary if year == `y'

local N = r(N)

quietly correlate gdp_primary ec_citypri if year == `y'

local r = r(rho)

local sumr = `sumr' + `r'

local count = `count' + 1

local t = `r' * sqrt(`N'-2) / sqrt(1-`r'^2)

local df `N' - 2

local p = 2 * (1 - ttail(`df', abs(`t')))

file write myresults "Year `y':\t r = `r',\t t = `t',\t p = `p'" _n

}

local meanr = `sumr' / `count'

local sumsq 0

forvalues y = 2005 / 2021 {

quietly correlate gdp_primary ec_citypri if year == `y'

local r = r(rho)

local sumsq = `sumsq' + (`r' - `meanr')^2

}

local sdr = sqrt(`sumsq' / (`count' - 1))

local cvr = (`sdr' / `meanr') * 100

di "Average correlation coefficient: `meanr', Coefficient of variation: `cvr'%"

file write myresults "Average correlation coefficient: `meanr', Coefficient of variation: `cvr'%" _n

file close myresults

**Results in Column (5)**

local path "C:\Users\PC\Desktop\Correlation coefficient\\results(secondary industry_FEC vs GDP).txt"

file open myresults using `"`path'"', write replace

local sumr 0

local count 0

forvalues y = 2005 / 2021 {

quietly sum gdp_second if year == `y'

local N = r(N)

quietly correlate gdp_second ec_citysec if year == `y'

local r = r(rho)

local sumr = `sumr' + `r'

local count = `count' + 1

local t = `r' * sqrt(`N'-2) / sqrt(1-`r'^2)

local df `N' - 2

local p = 2 * (1 - ttail(`df', abs(`t')))

file write myresults "Year `y':\t r = `r',\t t = `t',\t p = `p'" _n

}

local meanr = `sumr' / `count'

local sumsq 0

forvalues y = 2005 / 2021 {

quietly correlate gdp_second ec_citysec if year == `y'

local r = r(rho)

local sumsq = `sumsq' + (`r' - `meanr')^2

}

local sdr = sqrt(`sumsq' / (`count' - 1))

local cvr = (`sdr' / `meanr') * 100

di "Average correlation coefficient: `meanr', Coefficient of variation: `cvr'%"

file write myresults "Average correlation coefficient: `meanr', Coefficient of variation: `cvr'%" _n

file close myresults

**Results in Column (6)**

local path "C:\Users\PC\Desktop\Correlation coefficient\\results(tertiary industry_FEC vs GDP).txt"

file open myresults using `"`path'"', write replace

local sumr 0

local count 0

forvalues y = 2005 / 2021 {

quietly sum gdp_tertiary if year == `y'

local N = r(N)

quietly correlate gdp_tertiary ec_cityter if year == `y'

local r = r(rho)

local sumr = `sumr' + `r'

local count = `count' + 1

local t = `r' * sqrt(`N'-2) / sqrt(1-`r'^2)

local df `N' - 2

local p = 2 * (1 - ttail(`df', abs(`t')))

file write myresults "Year `y':\t r = `r',\t t = `t',\t p = `p'" _n

}

local meanr = `sumr' / `count'

local sumsq 0

forvalues y = 2005 / 2021 {

quietly correlate gdp_tertiary ec_cityter if year == `y'

local r = r(rho)

local sumsq = `sumsq' + (`r' - `meanr')^2

}

local sdr = sqrt(`sumsq' / (`count' - 1))

local cvr = (`sdr' / `meanr') * 100

di "Average correlation coefficient: `meanr', Coefficient of variation: `cvr'%"

file write myresults "Average correlation coefficient: `meanr', Coefficient of variation: `cvr'%" _n

file close myresults
