# Supplementary material for: A comprehensive city-level final energy consumption dataset including renewable energy for China, 2005–2021
Source: Sci Data. 2024 Jul 7;11:738. doi: 10.1038/s41597-024-03529-0 (PMC11228046; doi:10.1038/s41597-024-03529-0)
Supplement: Supplementary file 4 — The code of Table 3 [file 41597_2024_3529_MOESM4_ESM.docx]

use "C:\Users\PC\Desktop\城市能源平衡表编制\退修\code and data\Data_Table 3.dta", clear

**In order to match the balance panel, the city corresponding to the missing value is removed**

drop id0517 year0517 city0517 fe0517 ceads0517

egen mis = rowmiss(_all)

drop if mis

mdesc

asdoc pwcorr odiac0519 fe0519 if id0519==16,sig star(all)replace

asdoc pwcorr odiac0519 fe0519 if id0519==24,sig star(all)

asdoc pwcorr odiac0519 fe0519 if id0519==42,sig star(all)

asdoc pwcorr odiac0519 fe0519 if id0519==60,sig star(all)

asdoc pwcorr odiac0519 fe0519 if id0519==74,sig star(all)

asdoc pwcorr odiac0519 fe0519 if id0519==83,sig star(all)

asdoc pwcorr odiac0519 fe0519 if id0519==86,sig star(all)

asdoc pwcorr odiac0519 fe0519 if id0519==97,sig star(all)

asdoc pwcorr odiac0519 fe0519 if id0519==115,sig star(all)

asdoc pwcorr odiac0519 fe0519 if id0519==128,sig star(all)

asdoc pwcorr odiac0519 fe0519 if id0519==144,sig star(all)

asdoc pwcorr odiac0519 fe0519 if id0519==158,sig star(all)

asdoc pwcorr odiac0519 fe0519 if id0519==167,sig star(all)

asdoc pwcorr odiac0519 fe0519 if id0519==180,sig star(all)

asdoc pwcorr odiac0519 fe0519 if id0519==191,sig star(all)

asdoc pwcorr odiac0519 fe0519 if id0519==204,sig star(all)

asdoc pwcorr odiac0519 fe0519 if id0519==206,sig star(all)

asdoc pwcorr odiac0519 fe0519 if id0519==222,sig star(all)

asdoc pwcorr odiac0519 fe0519 if id0519==230,sig star(all)

asdoc pwcorr odiac0519 fe0519 if id0519==245,sig star(all)

asdoc pwcorr odiac0519 fe0519 if id0519==257,sig star(all)

asdoc pwcorr odiac0519 fe0519 if id0519==267,sig star(all)

asdoc pwcorr odiac0519 fe0519 if id0519==288,sig star(all)

asdoc pwcorr odiac0519 fe0519 if id0519==300,sig star(all)

asdoc pwcorr odiac0519 fe0519 if id0519==316,sig star(all)

asdoc pwcorr odiac0519 fe0519 if id0519==327,sig star(all)

use "C:\Users\PC\Desktop\城市能源平衡表编制\退修\code and data\Data_Table 3.dta", clear

**In order to match the balance panel, the city corresponding to the missing value is removed**

drop id0519 year0519 city0519 fe0519 odiac0519

egen mis = rowmiss(_all)

drop if mis

mdesc

asdoc pwcorr fe0517 ceads0517 if id0517==16,sig star(all)replace

asdoc pwcorr fe0517 ceads0517 if id0517==24,sig star(all)

asdoc pwcorr fe0517 ceads0517 if id0517==42,sig star(all)

asdoc pwcorr fe0517 ceads0517 if id0517==60,sig star(all)

asdoc pwcorr fe0517 ceads0517 if id0517==74,sig star(all)

asdoc pwcorr fe0517 ceads0517 if id0517==83,sig star(all)

asdoc pwcorr fe0517 ceads0517 if id0517==86,sig star(all)

asdoc pwcorr fe0517 ceads0517 if id0517==97,sig star(all)

asdoc pwcorr fe0517 ceads0517 if id0517==115,sig star(all)

asdoc pwcorr fe0517 ceads0517 if id0517==128,sig star(all)

asdoc pwcorr fe0517 ceads0517 if id0517==144,sig star(all)

asdoc pwcorr fe0517 ceads0517 if id0517==158,sig star(all)

asdoc pwcorr fe0517 ceads0517 if id0517==167,sig star(all)

asdoc pwcorr fe0517 ceads0517 if id0517==180,sig star(all)

asdoc pwcorr fe0517 ceads0517 if id0517==191,sig star(all)

asdoc pwcorr fe0517 ceads0517 if id0517==204,sig star(all)

asdoc pwcorr fe0517 ceads0517 if id0517==206,sig star(all)

asdoc pwcorr fe0517 ceads0517 if id0517==222,sig star(all)

asdoc pwcorr fe0517 ceads0517 if id0517==230,sig star(all)

asdoc pwcorr fe0517 ceads0517 if id0517==245,sig star(all)

asdoc pwcorr fe0517 ceads0517 if id0517==257,sig star(all)

asdoc pwcorr fe0517 ceads0517 if id0517==267,sig star(all)

asdoc pwcorr fe0517 ceads0517 if id0517==288,sig star(all)

asdoc pwcorr fe0517 ceads0517 if id0517==300,sig star(all)

asdoc pwcorr fe0517 ceads0517 if id0517==316,sig star(all)

asdoc pwcorr fe0517 ceads0517 if id0517==327,sig star(all)
